# Supplementary material for: Poly(Alkylene 2,5-Thiophenedicarboxylate) Polyesters: A New Class of Bio-Based High-Performance Polymers for Sustainable Packaging
Source: Polymers (Basel). 2021 Jul 27;13(15):2460. doi: 10.3390/polym13152460 (PMC8348809; doi:10.3390/polym13152460)
Supplement: Supplementary file 1 [file polymers-13-02460-s001.zip › polymers-1280435-supplementary.pdf]

Supplementary Materials

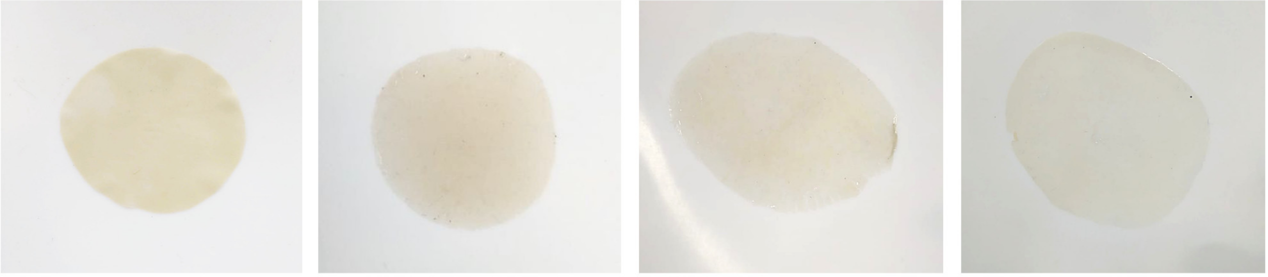

PPTF **PBTF** **PPeTF** **PHTF**

Figure S1. Pictures of compression moulded films.

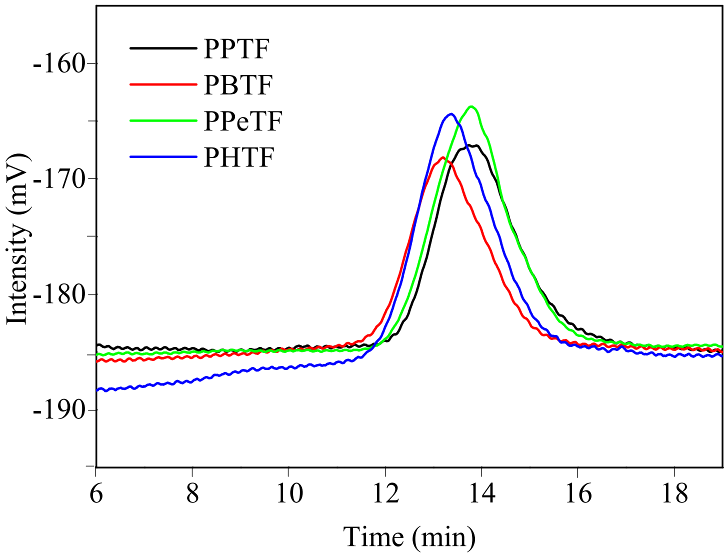

Figure S2. Chromatograms of the synthesized polymers.

Table S1. DSC data of the homopolymers under study in form of purified powders.

	**I scan**				**II scan**					
	T_g °C	Δc_p J/g°C	T_m °C	ΔH_m J/g	T_g °C	Δc_p J/g°C	T_{cc} °C	ΔH_{cc} J/g	T_m °C	ΔH_m J/g
PPTF	n.d	n.d	183	33	38	0.306	103	32	183	33
PBTF	25	0.130	147	33	25	0.320	93	23	147	23
PPeTF	4	0.128	67	29	4	0.324	-	-	-	-
PHTF	5	0.136	59<br>89	10<br>32	1	0.356	-	-	-	-

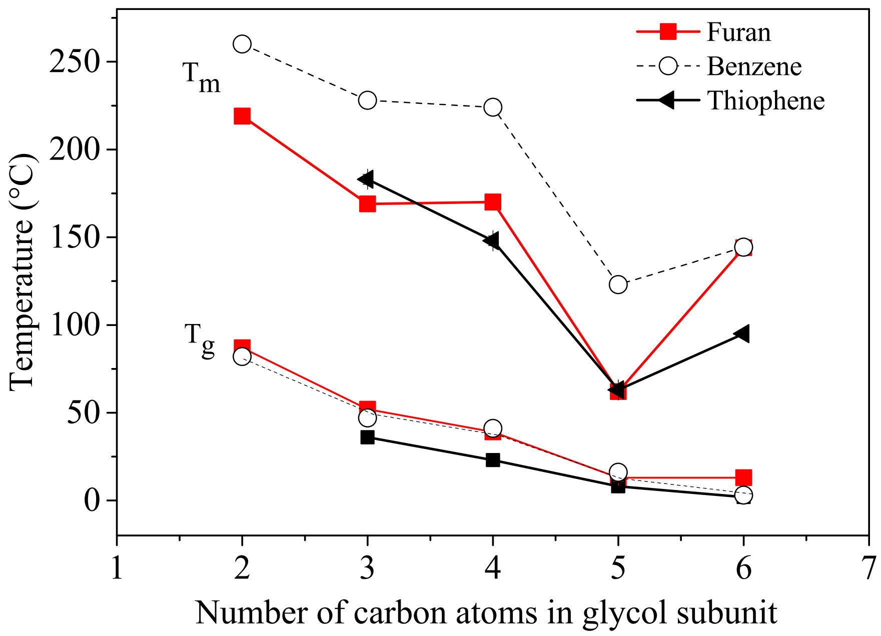

Figure S3. T_g and T_m trends as a function of glycolic subunit length for thiophene ring- (◀) containing polyesters compared to furan ring- (■) and benzene ring- (○) containing ones [29].
